# Global point prevalence study to determine the variations in aetiology, management and outcomes of pleural infection: a protocol for the international multicentre study of pleural infection (the INTERMITTENT Study)

**DOI:** 10.1136/bmjresp-2025-003288

**Published:** 2025-11-27

**Authors:** Steven P Walker, Francesca Gonnelli, Federico Mei, Uffe Bodtger, Jane A Shaw

**Affiliations:** 1 Academic Respiratory Unit, University of Bristol, Bristol, UK; 2 Biomedical Sciences and Public Health, Polytechnic University of Marche, Ancona, Italy; 3 Dep of Respiratory Medicine, Zealand University Hospital, Næstved, Denmark; 4 Department of Regional Health Research, University of Southern Denmark, Odense, Denmark; 5 Division of Immunology, Faculty of Medicine and Health Sciences, Stellenbosch University, Cape Town, South Africa

**Keywords:** Pleural Disease, Respiratory Infection, Tuberculosis

## Abstract

**Introduction:**

Pleural infection is a common problem associated with a high mortality. The aim of this study is to address current knowledge gaps in this condition by describing the global burden of disease and assessing the regional and seasonal variation in causative organisms, demographic characteristics, clinical presentation, comorbidities, pleural effusion characteristics, management strategies and outcomes. These data will inform future research and management guidelines.

**Methods and analysis:**

We will conduct an international multicentre point prevalence survey at two seasonal time points during the year. At each seasonal time point, data will be collected for 4 weeks on every episode of pleural infection presenting to the sites. Important demographic characteristics, medical history, clinical and imaging features, laboratory results, treatments and outcomes will be collected and analysed with a combination of descriptive and inferential statistics.

**Ethics and dissemination:**

The study will be performed in accordance with the Declaration of Helsinki and the International Conference on Harmonisation/Good Clinical Practice. This trial received ethical approval from the Comitato Etico Regione March, Italy, Prot. 2024 266. Institutional approvals will be acquired at all sites. All data will be treated confidentially and collected anonymously into an access-controlled electronic database. Results will be disseminated at conferences and in peer-reviewed publications.

**WHAT IS ALREADY KNOWN ON THIS TOPIC**

**WHAT THIS STUDY ADDS**

- This study will fill an important gap in the literature, using an existing international pleural research network.

**HOW THIS STUDY MIGHT AFFECT RESEARCH, PRACTICE OR POLICY**

- Future research and clinical practice around the world may be influenced by this study.

## Introduction

 Pleural effusion caused by an infectious organism is a common clinical condition which has potentially fatal outcomes. In Europe, up to 50% of people with pneumonia have a pleural effusion, which is associated with a 3–6-fold increase in mortality.^1^ In the USA, the annual incidence of hospitalised empyema is 11.7 per 100 000, with an in-hospital mortality of up to 7.2%, and a mean annual cost close to a billion USD.^2 3^ In England, the annual incidence rate of hospitalised empyema was 8.38 per 100 000 hospital admissions in 2017, with a mortality rate of approximately 14% over a 10-year period.^4^ In Africa, tuberculous effusions are highly prevalent, but—as with other low-middle-income regions—the epidemiology of pleural infection from other organisms is unknown.^5^ However, wherever you are in the world, the incidence of pleural infection seems to be increasing, and the burden on the healthcare system is substantial.^6^,^8^

The term ‘pleural infection’ describes a spectrum of conditions. These include a simple parapneumonic effusion, where an uninfected pleural exudate is associated with lung parenchymal infection; a complicated parapneumonic effusion, where the effusion has become infected and therefore requires drainage; and an empyema (derived from the Greek term ‘empyein’, meaning ‘to suppurate’), where there is frank pus in the pleural space.^9^

True pleural infection, which is often identified indirectly through pleural fluid pH or chemistry, is not uniformly managed. The age and comorbid status of the patient, the infecting organism, the presence of septae and loculations, a thick pleural peel or rind and the availability of treatment may all alter the management approach.^7^ While the RAPID score is a validated tool for predicting the outcome of pleural infection using some of these parameters (urea as an indicator of renal dysfunction, age, fluid purulence, community or nosocomial infection, and albumin level), because of the lack of high-quality evidence on many of the interventions which are available for pleural infection, common clinical practice still varies widely.^10^

These gaps in the evidence have been highlighted by a recent joint statement from the European Respiratory Society (ERS) and the European Society of Thoracic Surgeons.^7^ In their statement, they say that research efforts should focus on comprehensive characterisation of the burden of pleural infection in immunocompromised hosts and other neglected populations, and on documenting the microbiological patterns at a local level, on a global scale. Patients with pleural infection in low-middle-income countries are an important neglected population.

The International Multicentre Pleural Research Collaborative (IMPACT) is an ERS Clinical Research Collaboration (CRC) which consists of over 200 clinicians in more than 50 countries who are either experts in pleural disease or have a special interest in this field. Internal discussions within the network identified considerable regional variation in both the aetiology and management of pleural infection.

Building on this, IMPACT developed the protocol for the INTERMITTENT STUDY, which aims to fully describe international variations in the prevalence, aetiology and management of pleural infection.

The specific aims of INTERMITTENT are:

- To establish the burden of disease in terms of pleural infection cases in an international cohort and describe variations in burden by geographic location and seasonality.
- To establish the causative microorganisms of pleural infection in an international cohort and describe variations in microbiology by geographical location and seasonality.
- To describe the demographic characteristics, clinical presentation, comorbidities and pleural effusion characteristics of the patients with pleural infection in an international cohort, and describe variations by geographic location and seasonality.
- To describe management strategies of pleural infection in a diverse international cohort, stratified by the type of pleural infection and the geographic location.
- To associate management strategies with clinical outcomes, where possible, within the time frame of the project.

## Methods and analysis

This will be an international, multicentre point prevalence survey which will be coordinated by the existing IMPACT CRC network. A point-prevalence survey is a study designed for cross-sectional collection of information at multiple sites simultaneously, often used to report on antibiotic stewardship programmes.^11^ Sites within the global IMPACT CRC network will be invited to contribute data, with an emphasis on regions under-represented in the current literature. We will aim to recruit from at least four centres from each continent.

To capture the seasonal variation in pleural infection prevalence, there will be two data collection periods within 1 year, one in winter and one in summer. During the allocated timeframes, participating clinicians will collect data on all patients meeting the study definition of pleural infection seen in their centre.

### Inclusion criteria

INTERMITTENT will include any patient who underwent a pleural procedure for suspected pleural infection (see table 1), with pleural fluid culture and chemistry, during the data collection timeframe and within the previous 4 weeks.

**Table 1:** Study definitions in INTERMITTENT

Suspected pleural infection	Symptoms and signs suggestive of an infectious process, including, but not limited to, one or more of malaise, fever, chest pain, cough, raised white cell count, raised C reactive protein or other inflammatory/infectious marker in blood AND Chest imaging evidence of a pleural effusion
Chest imaging evidence of a pleural effusion	Chest radiograph, or chest CT, or thoracic ultrasound
Pleural procedure	Any procedure during which pleural fluid is aspirated from an effusion, in a patient fulfilling the criteria for suspected pleural infection. Pleural procedures where pleural fluid is aspirated but not sent for microbiological testing will not be included, except when the fluid is frank pus and another culture of blood or sputum has been sent.

Inpatients and outpatients may be included, and patients admitted to a high care or intensive care unit.

#### Exclusion criteria

- Patient is younger than 18 years.
- Patient has a suspected pleural infection as a result of recent thoracic trauma or surgery. Note on an exception: patients who have suspected pleural infection and a recent pleural biopsy will still be included, provided there was a pre-existing pleural effusion
- Patient is culture-positive for pulmonary or pleural Mycobacterium tuberculosis (TB)*.
- Known pleural malignancy (pleural fluid cytology positive for malignant cells or known thoracic malignancy or metastases).

*It is recognised that patients from areas of high incidence of TB, pleural infection can present similarly to TB pleuritis. Patients will not be excluded solely based on high pretest probability of TB. Patients who are included initially and eventually diagnosed with pleural TB will be dealt with separately in the analysis.

#### Data collection

Data collection will be over two 4-week periods within 12 months, from 1 to 28 February and 1 July to 28th July.

- Baseline variables of interest will be collected on the date that the potential pleural infection case is identified. Data on the outcome over the following 12 weeks will be collected retrospectively through routine clinical data collection tools.
- The following variables will be captured for each patient:
- Baseline demographics (age, sex at birth, geographic location, inpatient or outpatient status).
- Medical history and comorbidities (with a special focus on known risk factors for pleural infection such as frailty, diabetes mellitus and HIV).
- Details of the pleural procedure during which pleural fluid was aspirated (date, first or repeat procedure, complications, image-guided or blind).
- Ultrasound appearance of effusion at the time of diagnosis (simple or complex, loculated, pleural nodules or thickening, associated pericardial effusion, lung consolidation).
- Pleural fluid laboratory results at diagnostic aspiration (appearance, pH, biochemistry, cell count with differential, routine and mycobacterial microscopy, culture and sensitivity, any other tests).
- Other laboratory tests within 1 week of the diagnostic pleural aspiration (any other cultures, serum C reactive protein, procalcitonin, white cell count and differential, renal function, liver function).
- Evidence of viral coinfections or recent infections, such as respiratory pathogen PCR from nasopharyngeal swabs.
- Chest radiography and CT findings (effusion size and laterality, associated parenchymal abnormalities, pleural thickening or enhancement).
- Antibiotic regimen received and subjective clinical response.
- Intercostal drain insertion (date, size and type of drain, technique).
- Surgery details.
- Intrapleural enzyme therapy (regimen, date, response).
- Medical thoracoscopy (date, findings, interventions).
- RAPID score.
- Outcomes: duration of hospital stay; mortality; re-admission during follow-up; return to work within 30 days.
- Site pleural fluid collection and handling information (container selection for pleural fluid cultures, pleural fluid volumes sent for culture, centrifuging and concentration steps, culture method, method of sensitivity testing).

Data will be collected directly onto a specifically designed REDCap database hosted by the sponsor institution, University of Ancona, Italy. Clinicians will receive training on REDCap use in general and on the study database specifically before the initiation of the study. The built-in quality control processes in REDCap will be used during and after data collection to ensure maximal data completeness. Sites will keep confidential logs which link participant hospital identification numbers to a REDCap study number. Any information which might identify the participant will not be stored on the REDCap database. Each site will only be given access to view and edit their own entries onto the database using the ‘data access groups’ feature. After the quality control checks are completed, the database will be locked for editing.

### Sample size

INTERMITTENT is an observational study without an intervention or known effect size on which to base sample size calculations. Recruitment numbers will be dependent on the capacity of the sites as well as the factors which the study aims to describe such as local prevalence at the sites, population-level risk factors and seasonal variation.

### Analysis plan

The statistical analysis for the INTERMITTENT study will involve a detailed examination of the data to meet each of the specific study objectives. Descriptive and inferential statistical techniques will be employed, depending on the type of data and research questions addressed. Below is a breakdown of the planned statistical analyses for each objective:

Objective 1: To establish the burden of disease in terms of pleural infection cases in an international cohort and describe variations in burden by geographic location and seasonality.

- Descriptive statistics
  - Point prevalence: For each site and time period (winter and summer), the total number of pleural infection cases will be identified.
  - Geographical location: Total number of cases will also be stratified by country and region to examine each country’s contribution.
  - Seasonality: Comparisons of burden between winter and summer periods will be performed to explore seasonal variations.
- Inferential statistics
  - χ² test or Fisher’s exact test: These will be used to assess differences in prevalence across geographic regions and between the two data collection periods (seasonality).
  - Logistic regression: A logistic regression model will be used to adjust for potential confounding factors (such as patient demographics or comorbidities) and identify independent predictors of pleural infection prevalence across different regions and seasons.

Objective 2: To establish the causative micro-organisms of pleural infection in an international cohort and describe variations in microbiology by geographic location and seasonality.

- Descriptive statistics
  - The frequency of specific microorganisms (eg, *Staphylococcus aureus*, *Streptococcus pneumoniae*, *Mycobacterium tuberculosis*) isolated from pleural fluid cultures will be summarised.
  - Geographic and seasonal stratification of the data will show variations in the prevalence of specific micro-organisms.
- Inferential statistics
  - χ² test or Fisher’s exact test: These will be applied to compare the distribution of micro-organisms across different geographic regions and between winter and summer periods.
  - Multinomial logistic regression: This model will be employed to assess the association between geographical location, seasonality and the type of microorganism identified, adjusting for potential confounders such as antibiotic use and patient demographics.

Objective 3: To describe the demographic characteristics, clinical presentation, comorbidities and pleural effusion characteristics of the patients with pleural infection in an international cohort and describe variations by geographic location and seasonality.

- Descriptive statistics
  - Demographic characteristics (age, sex), clinical presentation (symptoms, severity of infection) and comorbidities (eg, diabetes, HIV, cancer) will be summarised for the entire cohort and stratified by geographic location and seasonality.
  - The characteristics of pleural effusion (eg, pH, glucose, protein, lactate dehydrogenase, cell count) will also be summarised and compared across regions and seasons.
- Inferential statistics
  - Analysis of variance or Kruskal-Wallis test: Continuous variables such as age, pleural fluid chemistry and blood markers will be compared across regions and seasons.
  - χ² test or Fisher’s exact test: Categorical variables such as comorbidities and clinical presentation will be analysed to detect differences across geographic regions and between seasons.
  - Multivariate regression models: These will be used to evaluate the relationship between demographic and clinical factors (eg, age, sex and comorbidities) and the characteristics of pleural infection, adjusting for geographic and seasonal variations.

Objective 4: To describe management strategies of pleural infection in a diverse international cohort, stratified by the type of pleural infection and geographic location.

- Descriptive statistics
  - The management strategies (eg, antibiotics used, duration of therapy, use of drainage procedures, surgery and intrapleural enzyme therapy) will be summarised and stratified by type of pleural infection (simple parapneumonic effusion, empyema) and by geographic location.
- Inferential statistics
  - χ² test or Fisher’s exact test: These will be used to compare the use of different management strategies across geographic regions.
  - Multivariable logistic regression: This will assess the factors associated with the choice of management strategy (eg, intercostal drainage vs medical management), adjusting for confounding factors such as pleural fluid characteristics, patient comorbidities and region.

Objective 5: To associate management strategies with clinical outcomes, where possible within the time frame of the project.

- Descriptive statistics
  - Clinical outcomes of interest (eg, duration of hospital stay, mortality, need for surgery, readmission, return to work) will be summarised for the overall cohort and stratified by management strategy (eg, antibiotic regimen, surgical intervention) and by geographical location.
- Inferential statistics
  - Survival analysis: Kaplan-Meier survival curves will be used to estimate time-to-event outcomes such as time to discharge, time to recovery and survival. The log-rank test will compare survival curves based on different management strategies.
  - Cox proportional hazards regression: This model will assess the association between management strategies (eg, type of antibiotic and the use of surgical intervention) and time-to-event outcomes (eg, survival and time to recovery), adjusting for covariates such as patient demographics, comorbidities and pleural fluid characteristics.
  - Multivariable logistic regression: This will be used to identify factors associated with binary outcomes such as mortality, readmission and the need for surgery. Independent variables will include management strategies, patient demographics, comorbidities and pleural fluid characteristics.

### Patient and public involvement

The Community Advisory Board of the Immunology Research Group of Stellenbosch University has been consulted on the study aims and design. Patient representatives have provided feedback on trial documents and will assist in the dissemination of trial results.

#### Participant information and informed consent

This study will collect anonymised routine clinical data at a single time point, with the remaining information collected retrospectively from hospital records. There will be no intervention, and the data collection will not affect any decisions regarding the patient’s management strategy. It is recommended, as this is routinely collected data, that sites request a waiver of written or verbal informed consent on a patient level. The investigators do, however, recognise that there will be variation in local practice and sites may need to obtain participant-informed consent. Each participating institution/site will be required to obtain institutional approval for the collection of these data. The sponsor will support participating sites with an application letter concerning the above waiver or an informed consent document as needed.

### Confidentiality

Data will be handled according to the ‘data handling and record keeping’ section above. Records which identify the patient or link them to the data collected by the study will be kept confidential, at the site they were collected from, and not be made publicly available. The deidentified data will be entered into a secure access-controlled REDCap database. Only the investigators responsible for data quality control and analysis, and specific collaborators will have access to these data. Data may be reviewed by representatives of the IRB/IEC, funding organisations or their representatives, and by others tasked with duties of monitoring and quality assurance. Research and clinical information relating to patients on the study may be shared with other researchers through lectures or publications, but the patients will not be identified by name. Any images (including chest radiograph, CT and ultrasound images) used for analysis or publication will be anonymised before use. All study-related documents with participants’ names, hospital numbers or addresses will be stored separately from their clinical information, and in secure access-controlled facilities at the sites.

### Data dissemination plan

The results of this study will be disseminated at international conferences, including the European Respiratory Society Congress, any local conferences of the investigators pending agreement by the group, and in publication format.

Authorship of publications will follow ICMJE guidelines. A plain English summary will be prepared and disseminated with the help of our ELF (European Lung Foundation) patient group.

## Data Availability

Data are available on reasonable request.
